# Effect of long non-coding RNA Gas5 on proliferation, migration, invasion and apoptosis of colorectal cancer HT-29 cell line

**DOI:** 10.1186/s12935-017-0478-7

**Published:** 2018-01-04

**Authors:** Jin Li, Yuan Wang, Cheng-Gong Zhang, Hai-Juan Xiao, Jun-Ming Hou, Jing-Dong He

**Affiliations:** 1 Department of Oncology, Huai’an First People’s Hospital, Nanjing Medical University, No. 6, Beijing Road West, Huai’an, 223300 People’s Republic of China; 2 Department of Oncology, Nanjing Medical University, Nanjing, 211166 People’s Republic of China

**Keywords:** Long non-coding RNA growth arrest-specific transcript 5, Colorectal cancer, HT-29, Proliferation, Migration, Invasion, Apoptosis

## Abstract

**Objective:**

This study aims to investigate the effect of long non-coding RNA (lncRNA) Gas5 on proliferation, migration, invasion and apoptosis of colorectal cancer (CRC) HT-29 cell line.

**Methods:**

CRC and normal tissues were collected and prepared from a total of 126 CRC patients, and normal intestinal epithelial cell line FHC and CRC cell lines (HCT-8, HT-29, HCT-116 and SW-480) were prepared. Gas5 expression was detected by quantitative reverse transcriptase-polymerase chain reaction. HT-29 cell line exhibiting the lowest Gas5 expression was selected for further experimentation and divided into blank, negative control and pcNDA-Gas5 groups. The cell counting kit-8 assay was used to test cell proliferation. Flow cytometry was applied to examine cell apoptosis. Transwell assay was performed to detect the migration and invasion of HT-29 cells. The mRNA and protein expression of factors in the classical proliferation (Akt/Erk) and apoptosis (caspase-9/caspase-3) pathways were detected.

**Results:**

Gas5 expression was lower in CRC tissues compared to the adjacent normal tissues, and is also lower in CRC cell lines than FHC cell line. Gas5 expression was associated with tumor size and TNM staging. Gas5 expression, distant metastasis, tumor differentiation and TNM staging were independent CRC prognostic factors. The results showed that elevated Gas5 expression inhibited proliferation, migration and invasion, but promoted apoptosis of CRC cells. Meanwhile, elevated Gas5 expression inhibited mRNA expression of Akt and Erk and protein expression of p-Akt and p-Erk, which promoted Casp9 mRNA and pho-Casp9 protein expression but inhibited Casp3 mRNA and pho-Casp3 protein expression.

**Conclusion:**

The findings indicated that overexpression of lncRNA Gas5 can inhibit the proliferation, migration and invasion but promote apoptosis of CRC cells.

## Introduction

Colorectal cancer (CRC) is the most common cause of cancer-related death across the world [1]. More than 50% CRC cases occur in developed countries [2]. Family history is one of the major factors for CRC [3]. Smoking [4], obesity [5] and older age [6] are also risk factors of CRC. A study showed that CRC incidence rates can be decreased by reducing risk factors and promoting healthy lifestyles [7]. For heterogeneous pathogenesis, CRC molecular maybe correlated to oxidative stress, splicing alterations, energy metabolism, microsatellite and chromosomal instability, hypermethylation of CpG islands, mutations in oncogenes and tumor suppressor genes, and impairment of different signaling pathways [8]. Recently, substantial improvements have been made in the diagnosis and treatment of CRC [9]. Although CRC incidence and mortality have decreased in recent years, the 5-year survival rate of CRC patients remains low [10] An early and accurate diagnosis as well as a precise evaluation of the survival post-operation would greatly improve the treatment for CRC [2]. A candidate molecular biomarker is required for patients with CRC.

Long non-coding RNAs (lncRNAs) have more than 200 nucleotides in length without protein-coding capacity [11]. LncRNAs are crucial for regulating the gene expression, which interact with major pathways, such as cell growth, proliferation, differentiation, and survival [12]. It can regulate the expression of genes in cis-formation and trans-acting formation [13]. The growth arrest-specific transcript 5 (Gas5) is encoded at 1q25 with 630 nucleotides in length, which is found to be upregulated in growth arrest [10]. LncRNA Gas5 can inhibit cell proliferation and promote apoptosis, which may provide the basis of its action as a tumor suppressor [14]. A study showed that lncRNA Gas5 may evaluate the surgical effects and prognosis for patients with breast cancer [15]. Also, another study provided evidence that overexpression of Gas5 can act as a tumor suppressor for renal carcinoma cell (RCC), providing a potentially valuable therapeutic approach for RCC [16]. However, the effects of lncRNA Gas5 on CRC treatment have rarely been reported. A previous study suggested that lncRNA Gas5 was an important tumor suppressor and could serve as a biomarker in CRC cells [17]. The study aims to explore the effect of lncRNA Gas5 on the proliferation, migration, invasion and apoptosis of human CRC cells.

## Materials and methods

### Study subjects

A total of 126 CRC patients who underwent surgical tumor resection from September 2011 to September 2013 at the Huai’an First People’s Hospital, Nanjing Medical University were included in this study. CRC and adjacent normal tissues were obtained and prepared from the patients, subsequently cooled in liquid nitrogen and stored at − 80 °C. The clinical data of patients were collected, including gender, age, tumor size, depth of invasion, lymph node metastasis, tumor differentiation, distant metastasis, tumor-node-metastasis (TNM) staging and tumor site. There were 74 males and 52 females, aged 31–72 years, with a calculated mean age of 55.13 ± 9.60 years. The tumor diameter was 3–8 cm, with an average of 5.32 cm. There were 28 cases with invasive depth in T1, 44 cases in T2, 35 cases in T3 and 19 cases in T4. There were 67 cases with lymph node metastasis, and 59 cases without lymph node metastases. There were 80 cases with distant metastases, and 46 cases without distant metastasis. In addition, there were 74 cases with well and moderately differentiated cells, and 52 cases with poor differentiated or undifferentiated cells. Patients with CRC in the right hemicolon were 58 cases, and 68 cases were with left hemicolon. Clinicopathological staging was in accordance with TNM staging system revised in 2002 as follows [18]: 60 cases in stage I and II, and 66 cases in stage III and IV. Inclusion criteria were CRC patients without preoperative chemotherapy, radiotherapy or other treatment for cancer, and CRC patients confirmed by histopathology. Exclusion criteria were patients with a history of other malignancies or other serious active diseases recently, and patients with drug hepatitis, alcoholic liver disease or autoimmune liver disease. Patients were followed up every 3 months at the first 2 years after surgery and followed up every 6 months from the 3rd year after surgery. The follow-up ended up in September 2016, and the overall survival of CRC patients was calculated. The study was approved by Huai’an First People’s Hospital, Nanjing Medical University, and signed informed consents were obtained from all participants.

### Quantitative reverse transcriptase-polymerase chain reaction (qRT-PCR)

Trizol reagent (Life Technologies, Grand Island, NY, USA) was used to extract RNA from CRC and adjacent normal tissues (50–100 mg) by high glucose DMEM with fetal bovine serum (FBS; 10%). Normal colonic epithelial cell line FHC and CRC cells were cultured at 37 °C in a humidified incubator with 5% CO_2_ in air and 90% humidity. RNA was extracted using the Trizol reagent from cells at the logarithmic phase of growth. Cells were selected from those with absorbance value 1.8–2.1 tested by spectrophotometer (A260/280 nm). Reverse transcription was performed in order to obtain cDNA in line with the instructions of the PrimeScript reverse transcriptase kit (Takara, Holdings Inc., Kyoto, Japan), and the cells were stored at − 20 °C.

The relative expression of Gas5 was detected in CRC and adjacent normal tissues, and in normal colonic epithelial cell lines FHC and 4 CRC cell lines (HCT-8, HT-29, HCT-116, SW-480) (forward 5′-TCGGCTTGACTACACTGTGT-3′, reverse 5′-GGAGGCTGAGGATCACTTGA-3′). β-actin was regarded as the reference gene (forward 5′-CATGGAATCCTGTGGCATCC-3′, reverse 5′-TGATCTTCATGGTGCTGGGA-3′). The reaction conditions were as follows: 50 °C for 2 min, 40 cycles of 95 °C for 10 min, 95 °C for 15 s and 58 °C for 60 s. All experiments were carried out three times. QRT-PCR, data acquisition and analysis were conducted using ABI7500 (Life Technologies, Grand Island, NY, USA). The relative expression of target genes was calculated by the 2^−△△Ct^ method.

### Cell culture and grouping

FHC and CRC cell lines (HCT-8, HT-29, HCT-116, and SW-480) were purchased from cell center in Shanghai Institutes for Biological Sciences, Chinese Academy of Sciences (Shanghai, China). All cell lines were tested for mycoplasma infection and identified using short tandem repeat (STR) by QIMR Berghofer Medical Research Institute [19]. It suggested that the study adopted stable cell lines without exogenous cell contamination, which met the requirements of the experiment. CRC cell lines (HCT-8, HT-29, HCT-116, and SW-480) and FHC cells were cultured in high glucose DMEM (Life Technologies, Grand Island, NY, USA) with 10% inactivated FBS at 37 °C in a humidified incubator with 5% CO_2_ in air. The medium was changed every 2–3 days according to its color. When cell confluence reached about 90%, the cells were passaged at a ratio of 1:3–1:5. Cells in the logarithmic phase of growth were selected for further experiments. HT-29 cells were selected for subsequent experiments for the lowest Gas5 mRNA expression in 4 CRC cell lines, and were divided into the blank group (HT-29), negative control (NC) group (transfected with the empty plasmid) and pcNDA-Gas5 group (transfected with pcNDA-Gas5).

### Construction of overexpressed Gas5 plasmid, cell transfection, and gene detection

HT-29 cells were collected. High glucose DMEM with 10% FBS (Life Technologies, Grand Island, NY, USA) was used to culture HT-29 cells in the logarithmic phase of growth at 37 °C in a humidified incubator with 5% CO_2_ in air and 90% humidity. Then, HT-29 cells were transfected in accordance with the instructions of lipofectamine 2000 (Invitrogen Inc., Carlsbad, CA, USA). HT-29 cells were inoculated in a 6-well plate containing 1 × 10^5^ cells per well, and transfected after cell confluence reached 50–70%. Overexpressed Gas5 plasmid was constructed in the early stage of this experiment. Primers were as follows: GAS5-XhoI-F, 5′-ccgctcgag TTTCGAGGTAGGAGTCGACTCCTGTG-3′; GAS5-BamHI-R, 5′-cgcggatcc TTTTTTTTTTTTTTTTTTTGTATTGCAAA-3′. These primers contained restriction sites for XhoI and BamHI (underline). Full-length fragment of Gas5 cDNA was amplified by RT-PCR. A reaction system of 25 μL contained 2 mM dNTP mix, 2.5 μL 10× KOD buffer, 1.5 μL 25 mM MgSO_4_, 0.5 μL synthetic template, 0.3 μL PCR forward primers, 0.3 μL PCR reverse primers, 0.3 μL KOD Plus Neo, and 17.1 μL RNA-free water. PCR reaction conditions were as follows: 3 min of pre-denaturation at 94 °C, followed by 30 cycles (98 °C for 15 s, 58 °C for 15 s, 95 °C for 30 s). The products were stored at 16 °C, separated and purified. After double enzyme digestion, the PCR products were inserted into the downstream promoter of pcDNA vector carrying cytomegalovirus (CMV) by T4 DNA ligase to construct a pcDNA-Gas5 overexpression plasmid and empty plasmid. The recombinant plasmid was then transformed into DH5α competent cell for sequencing verification [20]. Interference series (50 nmol) were taken from each gene for transfection according to Lipofectamine 2000 (Life Technologies, Grand Island, NY, USA), and after 4–6 h, DMEM with 10% FBS was used for cultivation for another 48 h. TaqMan^®^ Reverse Transcription Kit and TaqMan^®^ 2× Universal PCR Master Mix (Applied Biosystems, Grand Island, NY, USA) were used to detect the expression levels of Gas5 and proliferation and apoptosis associated genes (Akt, Erk, Casp3, Casp-9) in HT-29 cells in the blank group. The primer sequences were as follows: Akt, forward 5′-CGGAATTCATGAGCGACGTGGCTATTGTG-3′, reverse 5′-GGCTCGAGTCAGGCCGTGCCGCTGG-3′; Erk, forward 5′-CGGAATTCCTGTTAACATTGTCATTCC-3′, reverse 5′CGAAGCTTTTCATCATTGAAGATAGCG-3′; Casp3, forward 5′-TGACCTATCCTGCCCTCA-3′, reverse 5′-TGTCCTGCCTCACTACTGTCC-3′; Casp9, forward 5′-GCCATACAAACTGGATGATGAC-3′, reverse 5′-CACTGCTCAAAGATGTCGTCCA-3′. The method and specific steps were the same as above. After CRC cell line HT-29 transfected with empty plasmid and Gas5 gene for 48 h, the cells were collected to detect the transient expression of Gas5 Akt, Erk, Casp3 and Casp-9 by total RNA extraction, reverse transcription and qRT-PCR.

### Western blot for the detection of Gas5 protein and proliferation and apoptosis related proteins [21–23]

After transfection for 48 h, the cells were lysed for 30 min on ice using radioimmunoprecipitation (RIPA) protein lysate (PS0013, Leagene Biological Technology Co. Ltd., Beijing, Chinese) and centrifuged for 20 min at 4 °C at a rate of 12,000 rpm. The supernatant was collected and sub-packed. Total protein content was determined using the bicinchoninic acid (BCA) kit (23250, Thermo Fisher Scientific, Shanghai, China) and placed in a refrigerator − 80 °C after sub-packing. 50 µg protein was selected in each group, boiled for 10 min with protein denaturant (38249090, Shanghai Shisheng Sibas advanced Technology Co., Ltd., Shanghai, China), then separated by sodium dodecyl sulfate polyacrylamide gel electrophoresis (SDS-PAGE), and transferred to a nitrocellulose (NC) filter by electrophoretic transfer. Subsequently, the NC filter was sealed for 12 h at 4 °C in poly(butylene succinate–terephthalate) (PBST) containing 10% skimmed milk, rinsed three times with PBST (5 min per wash), correspondingly added primary antibodies (rabbit anti-human Gas5, diluted at a ratio of 1:500, ab136249; rabbit anti-human p-AKT, diluted at a ratio of 1:1000, ab8932; rabbit anti-human p-Erk1/2, diluted at a ratio of 1:1000, ab40658; rabbit anti-human pho-Casp3, diluted at a ratio of 1:1000, ab59425; rabbit anti-human pho-Casp-9, diluted at a ratio of 1:1000, ab135544; Abcam Inc., Cambridge, MA, USA), incubated 2 h at 37 °C, and rinsed three times with PBST (10 min per wash). Next, the horseradish peroxidase labeled goat-anti-rabbit IgG (diluted at a ratio of 1:1000, DF109489, Yaoyun Biotechnology Co., Ltd., Shanghai, China) was added to the cells followed by incubation for 2 h at 37 °C. Following rinsing with PBST three times (10 min per wash), chemiluminescence method (ECL kit, 36208ES60, Amersham Life Sciences, Arlington Hts, Illinois, USA) was applied for developing a film and ImageJ software for quantitative analysis. With glyceraldehyde phosphate dehydrogenase (GAPDH) serving as the reference, the gray value ratio of the target strip to the inner reference band was regarded as the relative expression levels of protein. Each sample was set with three repetitions.

### Cell counting kit-8 (CCK8) assay

The cells in the blank group, NC group and pcDNA-Gas5 group were seeded in a 96-well plate containing 5 × 10^3^ cells per well, respectively, and each well was supplemented with DMEM (100 µL). Six wells in each group at the 12th, 24th, 48th, 72nd and 96th h time periods were incubated for 1.5 h with 10 µL CCK8 solution. The absorbance (optical density [OD] value) was detected at a wavelength of 450 nm by enzyme-linked immunosorbent assay. Cell viability was calculated according to the following formula: [(OD in the experimental group − OD in the bank group)/(OD in the NC group − OD in the bank group)] × 100%.

### Flow cytometry

After transfection for 48 h, the cells in the blank, NC and pcDNA-Gas5 groups were digested using trypsin (2.5 g/L) and centrifuged for 5 min at a rate of 1000 rpm. The supernatant was discarded, and the cells were rinsed twice with cold PBS, suspended using ethanol (75%) and fixed for 12 h at 4 °C. The re-suspended HT-29 cells were mixed gently with PI (200 µL), preserved for 15 min at room temperature deprived of light, and then supplemented with 300 µL binding buffer. Flow cytometry was performed for cell apoptosis analysis. The experiment was repeated three times.

### Transwell assay

After transfection for 24 h, the cells were digested with trypsin, washed by serum-free medium, and re-suspended by DMEM medium (containing 1% FBS) to adjust the cell density to 1 × 10^6^ cells/mL. Cell suspension (100 µL, about 1 × 10^4^ cells) was added to the upper chamber, and 500 µL DMEM medium (with 10% FBS) was added into the lower chamber to culture the cells for 16 h at 37 °C in a humidified incubator with 5% CO_2_ in air. The culture medium was discarded and the cells were fixed with 95% ethanol at room temperature for 30 min. The cells were next stained by 0.1% crystal violet for 30 min and rinsed with water. The cells that did not migrate were gently wiped out with a cotton bud. Cells that migrated to the lower chamber were counted under a microscope (×400). Ten visual fields were selected randomly from each well, and the average value was adopted. The experiment was repeated three times.

Polycarbonate membrane of the upper chamber in the 6-well plate (Corning Glass Works, Corning, NY., USA) was supplemented with 60–80 µL diluted Mattrigel matrix (3.9 µg/µL, Becton, Dickinson and Company, San Jose, CA, USA), and matrigel was polymerized into the gel at 37 °C for 30 min. Matrigel glue (15 µg/mL) was evenly placed on the membrane of Transwell chamber for freezing, and then hydrated for 30 min at 37 °C with serum-free DMEM. About 1 × 10^5^ HT-29 cells in the NC and pcDNA-Gas5 groups and 200 µL suspensions from the blank group were inoculated into upper chambers, separately. Lower chambers were added with 600 µL DMEM containing 10% FBS. After being immersed in a water bath for 24 h at 37 °C, the chambers were removed, and a cotton bud was used to wipe the cells that did not pass through the microporous membrane. Membranes were fixed with methanol for 20 min, and stained by crystal violet for 10 min. The cells were counted in 10 visual fields under a microscope (×400), and the average value was adopted. The experiment was repeated three times.

### Statistical analysis

Statistical analyses were performed using the SPSS 21.0 software. Data with normal distribution were represented as mean ± standard deviation (SD). Measurement data were compared using the *t* test or variance analysis. Cases lost-to-follow-up and survival cases at the end of follow-up period were regarded as censored data. Grade data were analyzed by the nonparametric rank sum test and the survival rate was measured by the log-rank test of Kaplan–Meier survival analysis. COX regression was used to evaluate the effect of Gas5 expression and clinical parameters on the total survival rate of patients. *P* < 0.05 was considered to be statistically significant.

## Results

### Expression of Gas5 in CRC tissues and CRC cell lines

**Fig. 1:**
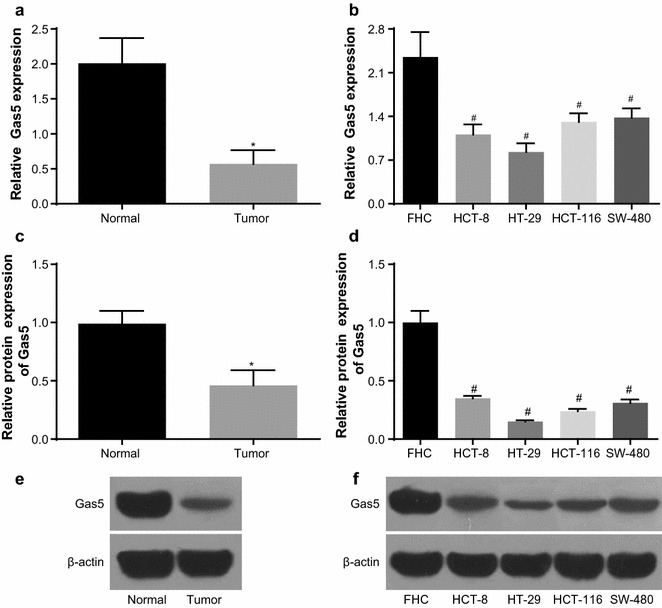
Gas5 relative expression in CRC tissues and adjacent tissues, FHC and CRC cell lines. **a** Gas5 relative mRNA expression in CRC tissues and adjacent tissues; **b** Gas5 relative mRNA expression in FHC and CRC cell lines; **c** Gas5 relative protein expression in CRC tissues and adjacent tissues; **d** Gas5 relative protein expression in FHC and CRC cell lines; **e** Gas5 protein band in CRC tissues and adjacent tissues; **f** Gas5 protein band in FHC and CRC cell lines. *P < 0.05 compared with the adjacent normal tissues; ^#^*P* < 0.05 compared with FHC cell line. *FHC* normal human intestinal epithelial cell line, *CRC* cell lines = HCT-8, HT-29, HCT-116, SW-480; *CRC* colorectal cancer

The relative mRNA expression of Gas5 in CRC tissues was 0.57 ± 0.20, which was evidently lower compared to the adjacent normal tissues (2.01 ± 0.36) (*P* < 0.05, Fig. 1a). Gas5 mRNA expression in CRC cell lines (HCT-8, HT-29, HCT-116, SW-480) was 1.11 ± 0.16, 0.83 ± 0.14, 1.31 ± 0.14 and 1.38 ± 0.15, lower compared to the normal colonic epithelial cell line FHC (2.35 ± 0.40) (*P* < 0.05, Fig. 1b). Protein expression of Gas5 in adjacent normal tissues was significantly higher compared to the CRC tissues (*P* < 0.05, Fig. 1c, e), and Gas5 protein expression in CRC cell lines was lower compared to the normal colonic epithelial cell line (*P* < 0.05, Fig. 1d, f). HT-29 cell line with the lowest mRNA and protein expression of Gas5 was selected for further experimentation.

### Correlation of Gas5 expression with clinicopathological features of patients with CRC

As shown in Table 1, Gas5 mRNA and protein expression in CRC tissues was associated with tumor size and TNM staging (*P* < 0.05). The Gas5 mRNA and protein expression was higher whereas the tumor diameter was smaller and TNM staging was lower. Gas5 mRNA and protein expression was not correlated to age, gender, tumor site, tumor differentiation or lymph node metastasis (*P* > 0.05).

**Table 1:** Correlation of Gas5 expression with clinicopathological features of patients with CRC

Clinicopathological features	N	Gas5 mRNA expression	*P*	Gas5 protein expression	*P*
Age (years)					
≤ 60	79	0.59 ± 0.20	0.162	0.46 ± 0.14	0.691
> 60	47	0.54 ± 0.18		0.47 ± 0.13	
Gender					
Male	74	0.56 ± 0.20	0.582	0.45 ± 0.11	0.214
Female	52	0.58 ± 0.20		0.48 ± 0.16	
Tumor size (cm)					
≤ 5	45	0.64 ± 0.19	0.002	0.53 ± 0.15	< 0.001
> 5	81	0.53 ± 0.19		0.42 ± 0.10	
Invasive depth					
T1	28	0.62 ± 0.22	0.185	0.47 ± 0.14	0.053
T2	44	0.59 ± 0.18		0.50 ± 0.15	
T3	35	0.54 ± 0.21		0.44 ± 0.11	
T4	19	0.50 ± 0.16		0.41 ± 0.10	
Lymph node metastasis					
With	67	0.55 ± 0.17	0.153	0.45 ± 0.14	0.202
Without	59	0.60 ± 0.22		0.48 ± 0.12	
Distant metastasis					
With	80	0.55 ± 0.21	0.107	0.45 ± 0.12	0.103
Without	46	0.61 ± 0.18		0.49 ± 0.15	
Tumor differentiation					
Well or moderate differentiation	74	0.55 ± 0.21	0.158	0.47 ± 0.13	0.412
Poor or no differentiation	52	0.60 ± 0.18		0.45 ± 0.14	
TNM staging					
I–II	60	0.66 ± 0.19	< 0.001	0.49 ± 0.12	< 0.034
III–IV	66	0.49 ± 0.17		0.44 ± 0.14	
Tumor site					
Right hemicolon	68	0.58 ± 0.20	0.577	0.46 ± 0.15	0.684
Left hemicolon	58	0.56 ± 0.20		0.47 ± 0.12	

*Gas5* growth arrest-specific transcript 5, *CRC* colorectal cancer, *TNM* tumor-node-metastasis

### Correlation of Gas5 expression with the survival of CRC patients

**Fig. 2:**
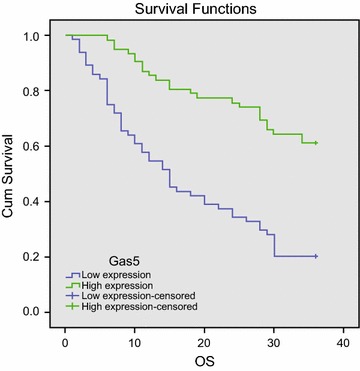
Correlation between Gas5 expression and the survival time of patients with CRC. *CRC* colorectal cancer

A total of 126 patients with CRC were divided into low Gas5 expression and high Gas5 expression groups according to the median expression of Gas5 (0.57). Kaplan–Meier analysis showed that the survival time in the low Gas5 expression group was significantly lower compared to the high expression Gas5 group (*P* < 0.05). The median survival times of Gas5 in the high and low Gas5 expression groups were 29 and 24 months, respectively (Fig. 2). After univariate analysis, the difference factors were included in the Cox model. Univariate analysis and COX analysis showed that lymph node metastasis, Gas5 expression, TNM staging, distant metastasis and tumor differentiation were closely related to the prognosis of CRC patients (*P* < 0.05). Multivariate COX regression analysis showed that the Gas5 expression, distant metastasis, tumor differentiation and TNM staging could serve as independent prognostic factors in patients with CRC (Table 2).

**Table 2:** Correlation of Gas5 expression and clinicopathological features with the overall survival rate of CRC patients

Factors	Univariate analysis		Multivariate analysis	
	Chi square	*P*	HR (95% CI)	*P*
Tumor site	2.24	0.135		
Gender	0.21	0.650		
Age	1.81	0.178		
Tumor size	1.53	0.216		
Invasive depth	5.64	0.303		
Lymph node metastasis	4.43	0.035	1.50 (0.93–2.43)	0.099
Gas5	27.13	< 0.001	0.52 (0.03–0.91)	0.022
Distant metastasis	20.14	< 0.001	2.76 (1.54–4.93)	0.001
Tumor differentiation	5.80	0.016	2.00 (1.25–3.20)	0.004
TNM staging	34.67	< 0.001	2.81 (1.56–5.04)	0.001

*Gas5* growth arrest-specific transcript 5, *CRC* colorectal cancer, *TNM* tumor-node-metastasis

### Gas5 expression in HT-29 cell line among the blank, NC and pcDNA-Gas5 groups

**Fig. 3:**
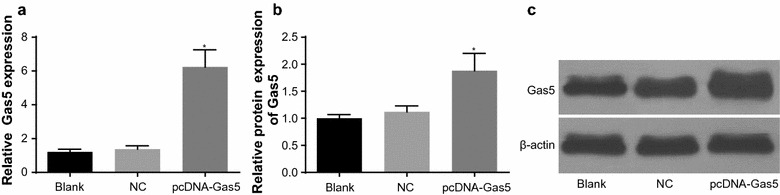
The relative expression of Gas5 in HT-29 cells among the blank, NC and pcDNA-Gas5 groups. **a** relative mRNA expression of Gas5 using qRT-PCR; **b** relative protein expression of Gas5; **c** gray value of Gas5 using Western blotting (1 represents the blank group; 2 the NC group; and 3 the pcDNA-Gas5 group). *P < 0.05 compared with the blank and NC groups. *NC* negative control, *qRT-PCR* quantitative reverse transcriptase-polymerase chain reaction

After HT-29 cell lines were transfected with pcDNA-Gas5 for 48 h, qRT-PCR was used to detect the Gas5 expression in the pcDNA-Gas5, blank and NC groups. The Gas5 relative expression had no distinguishing difference between the NC group (1.38 ± 0.19) and blank group (1.21 ± 0.16) (*P* > 0.05). The relative expression in the pcDNA-Gas5 group was 6.24 ± 1.02, higher than that in the blank and NC groups (*P* < 0.05). Compared with the blank and NC groups, Gas5 protein expression in the pcDNA-Gas5 group increased significantly, indicating the Gas5 expression of HT-29 cells was significantly upregulated after the transfection with pcDNA-Gas5 (Fig. 3).

### Gas5 inhibited the proliferation of HT-29 cells

**Fig. 4:**
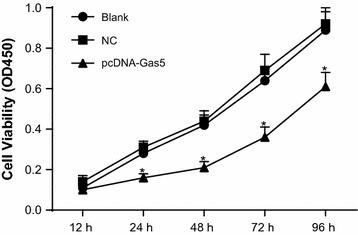
Cell viability of HT-29 cell tested by the CCK8 assay. *P < 0.05 compared with the blank and NC groups. *NC* negative control, *OD* optical density

As it was shown in Fig. 4, compared with the blank group, there were no significant differences in the NC group (*P* > 0.05). The growth of HT-29 cells was slower in the pcDNA-Gas5 group compared to the blank and NC groups at the 24th, 48th, 72nd and 96th h time periods (*P* < 0.05).

### Gas5 promoted the apoptosis of HT-29 cells

**Fig. 5:**
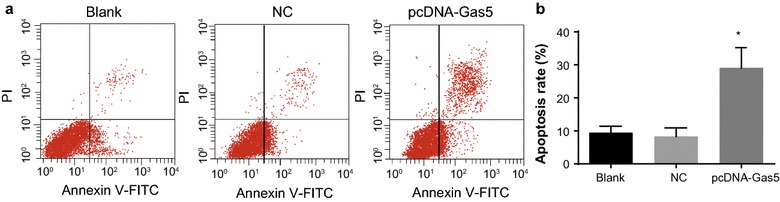
HT-29 cell apoptosis. **a** cell apoptosis results tested by flow cytometry; **b** comparison of cell apoptosis rate in the blank, NC and pcDNA-Gas5 groups. *P < 0.05 compared with the blank and NC groups. *NC* negative control

There was no marked difference in the apoptosis rate between the blank and NC groups (*P* > 0.05). Compared with the blank [(9.48 ± 1.92)%] and NC groups [(8.33 ± 2.57)%], the apoptosis rate in the pcDNA-Gas5 group was markedly increased [(29.10 ± 6.12)%] (*P* < 0.05, Fig. 5).

### Elevated Gas5 expression inhibited the migration and invasion of HT-29 cells

**Fig. 6:**
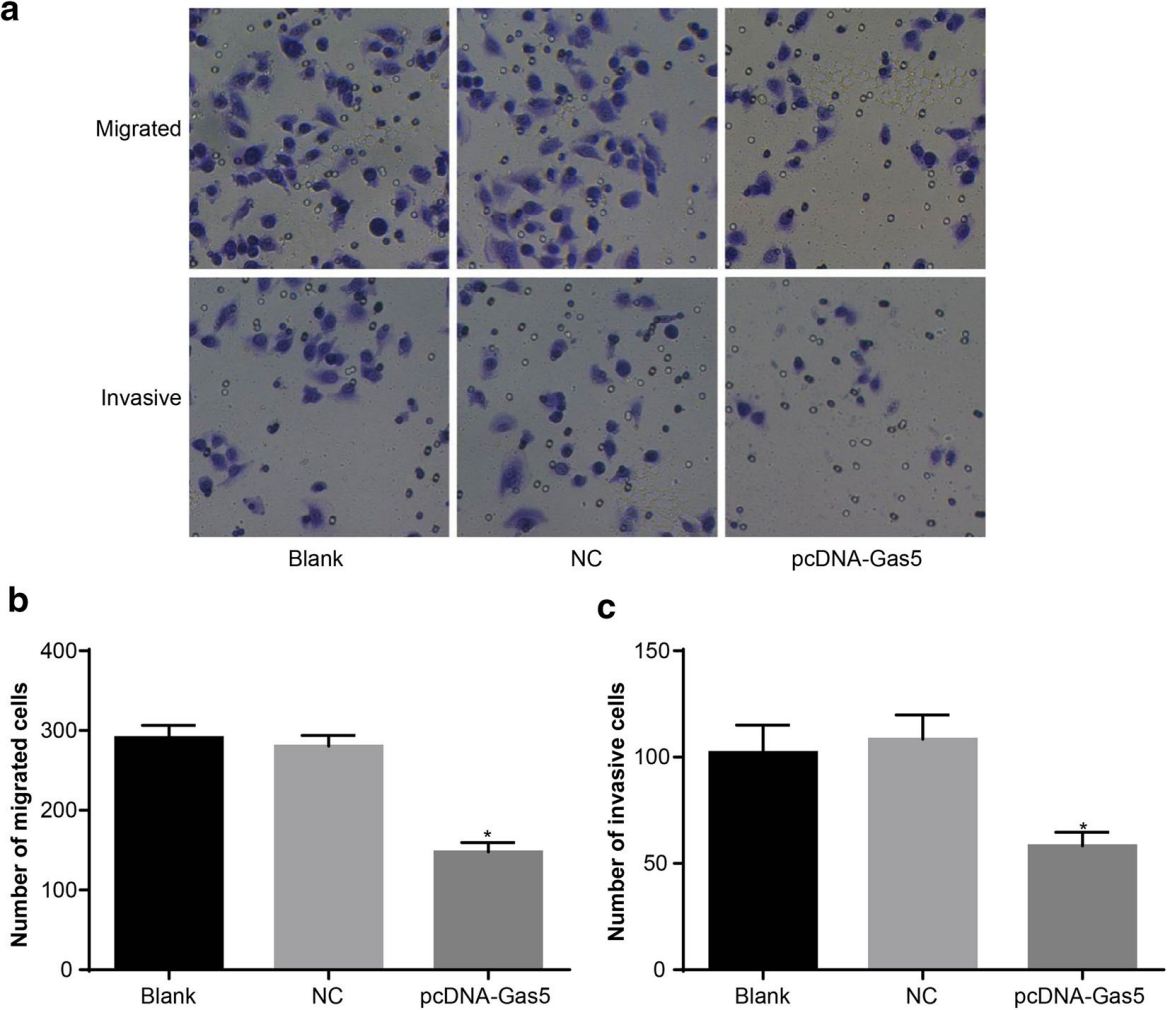
HT-29 cell migration and invasion tested by Transwell. **a** Transwell was used to detect the cell migration and invasion (× 400); **b** cell migration ability in each group; **c** cell invasive ability in each group. *P < 0.05 compared with the blank and NC groups. *NC* negative control

The number of migrated cells in the blank group and NC group was 291.32 ± 15.34 and 280.56 ± 13.29, respectively. There were no significant differences between the blank and NC groups (*P* > 0.05). The number of migrated cells in the pcDNA-Gas5 group was 147.74 ± 11.61, which was lower compared to the blank and NC groups (*P* < 0.05). These results indicated that an elevated Gas5 expression could inhibit the migration ability of HT-29 cells. After cells were transfected for 48 h, there were no significant differences in the number of invasive cells between the blank group (102.21 ± 12.87) and NC group (108.43 ± 11.38) (*P* > 0.05). The number of invasive cells in the pcDNA-Gas5 group was 58.34 ± 6.39, lower compared to the blank and NC groups (*P* < 0.05). These results indicated that an elevated Gas5 expression could inhibit the invasion ability of HT-29 cells (Fig. 6).

### The mRNA and protein expression of related genes and proteins in classical proliferation (Akt/Erk) and apoptosis (caspase-9/caspase-3) pathways

**Fig. 7:**
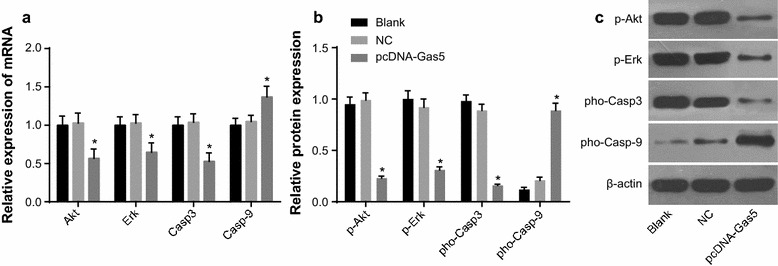
Expression of proliferation and apoptosis associated genes and proteins. **a** mRNA expression of Akt, Erk, Casp3 and Casp9 using qRT-PCR; **b** protein expression of p-Akt, p-Erk, pho-Casp3 and pho-Casp9; **c** gray value of p-Akt, p-Erk, pho-Casp3 and pho-Casp9 using Western blotting. *P < 0.05 compared with the blank and NC groups. *NC* negative control, *qRT-PCR* quantitative reverse transcriptase-polymerase chain reaction

There were no statistical differences between the blank and NC groups (*P* > 0.05). Compared with the blank and NC groups, the relative expression of Casp9 mRNA and pho-Casp9 protein in the pcDNA-Gas5 group was increased, and the relative expression of Akt, Erk and Casp3 mRNA and p-Akt, p-Erk and pho-Casp3 protein was decreased (*P* < 0.05). It suggested that an elevated Gas5 expression could affect the expression of proliferation and apoptosis associated genes and proteins (Fig. 7).

## Discussion

CRC with heterogeneous outcomes and drug responses is one of the malignant cancers across the world [24]. It has been reported that the 1-year survival rate of CRC patients is 83.2%, and the 5-year survival rate is 64.3%, while the survival rate gradually decreases to 57.6% 10 years post-diagnosis [25]. The available treatments for CRC present with various unfavorable side effects [26]. Functional studies have validated that lncRNA Gas5, acting as a tumor repressor could potentially inhibit proliferation and promote apoptosis of several cell types [14]. However, the underlying mechanism of Gas5 in the treatment of CRC remains to be unclear. Thereby, in this study, we carried out experiments on the hypothesis that Gas5 could serve as a CRC prognostic marker and therapeutic target to identify the effect of Gas5 on the development of CRC.

Firstly, the expression of Gas5 in CRC tissues was lower compared to the adjacent normal tissues. Moreover, the expression of Gas5 was upregulated in conventional CRC cell lines in comparison to normal intestinal epithelial cell line FHC. Gas5 is a potent inhibitor of glucocorticoid receptors (GR) by blocking GR from being activated and preventing GR from regulating the target genes’ transcription [27]. Gas5 expression is rapidly reduced in some cancers, such as gastric cancer and renal carcinoma [16, 28]. A previous study illustrated that Gas5 was markedly downregulated in tissues, serum and cell lines of CRC patients, and negatively related to the cytokine expression in the mouse model of colitis-associated cancer [29]. Furthermore, Kaplan–Meier results indicated shorter survival time in patients with lower Gas5 expression compared to the patients with higher Gas5 expression. A study using the log-rank test showed that cervical cancer patients with a low Gas5 expression tended to have a shorter overall survival time compared to those with a high Gas5 expression [30].

In addition, we found that Gas5 expression exerted a negative control in tumor size and TNM staging. A small-sized tumor diameter and early TNM staging resulted in a higher Gas5 expression. Gas5 expression has been reported to be related to clinicopathological characteristics, like tumor size, staging, lymph node metastasis and invasion [14]. A study suggested that Gas5 expression was significantly lower in gastric cancer tissues compared to normal tissues, and Gas5 with lower expression was associated with larger tumor diameter and a more advanced clinical stage of gastric cancer [31]. Additionally, a study revealed that compared with adjacent noncancerous tissues, Gas5 expression was diminished in non-small cell lung cancer tissues, which was highly related to TNM staging and tumor size [32]. Furthermore, multivariate COX regression analysis in the present study showed that Gas5 expression, distant metastasis, tumor differentiation and TNM staging could serve as independent prognostic factors for CRC. A previous study showed that distant metastasis was a major challenging for the advanced CRC treatment, which resulted in a low survival rate of CRC patients [33]. Evidences showed that high tumor differentiation was a potential indicator of poor prognosis in colonic adenocarcinomas and CRC [34, 35].

HT-29 cells with the lowest Gas5 expression were selected for further experimentation and we found that the expression of Gas5 was significantly upregulated after the HT-29 cells were transfected with pcDNA-Gas5. And the in-depth experiment showed that elevated Gas5 expression could negatively affect the expression of signaling pathway-related proteins. It was observed that Gas5 overexpression could contribute to inhibiting the proliferation, migration and invasion and promoting apoptosis of CRC cells, further solidifying our findings and successfully accomplishing the aim of this study. Preliminary functional exploration revealed that the proliferation, invasion and migration abilities of renal carcinoma cells were repressed by an upregulated Gas5 expression [16]. Likewise, a previous study revealed that overexpression of Gas5 inhibited cell proliferation of CRC in vitro and vivo, and showed that Gas5 may serve as an appropriate prognostic biomarker for CRC [10].

## Conclusion

In conclusion, our results demonstrate that Gas5 plays a crucial role in the clinicopathological features and prognosis of CRC, which may provide significant clues for further clinical practice of CRC. However, differences among populations affecting Gas5 gene polymorphisms may serve as the limitations to this study. Thus, further research and studies need be carried out based on a larger sample size. Nonetheless, these findings provide a potential value for Gas5 in the clinical treatment of CRC patients.

## Figures and Tables

**Fig. 8:**
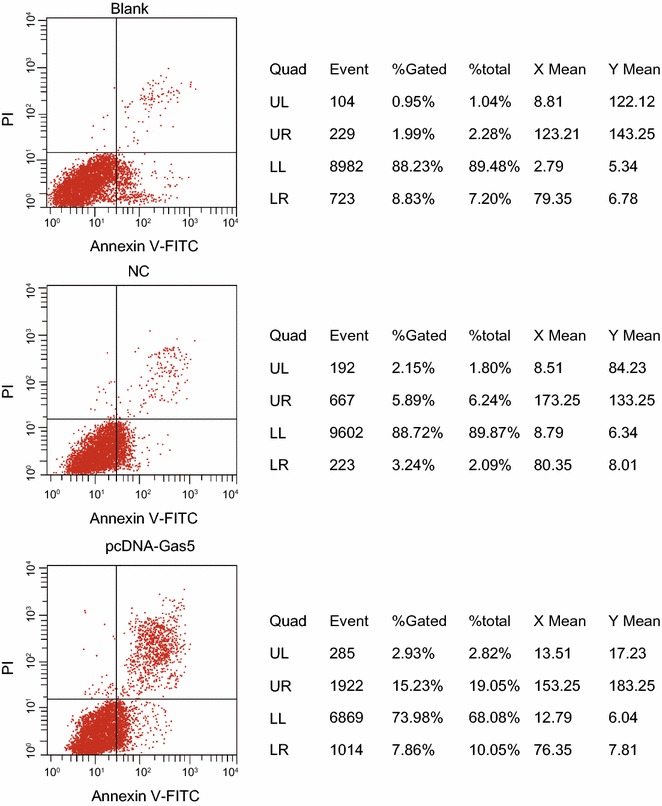
HT-29 cell apoptosis in each group detected by flow cytometry

## Appendix

See Fig. 8.

## Abbreviations

CRC: colorectal cancer
NC: negative control
CCK8: cell counting kit-8
