# Correction to: Effect of long non-coding RNA Gas5 on proliferation, migration, invasion and apoptosis of colorectal cancer HT-29 cell line

**DOI:** 10.1186/s12935-018-0510-6

**Published:** 2018-01-31

**Authors:** Jin Li, Yuan Wang, Cheng-Gong Zhang, Han-Juan Xiao, Jun-Ming Hu, Jing-Dong He

**Affiliations:** 1 Department of Oncology, Huai’an First People’s Hospital, Nanjing Medical University, No. 6, Beijing Road West, Huai’an, 223300 People’s Republic of China; 2 Department of Oncology, Nanjing Medical University, Nanjing, 211166 People’s Republic of China

## Correction to: Cancer Cell Int (2018) 18:4 10.1186/s12935-017-0478-7

After publication of the original article [1] it was noted that the names of two authors, Han-Juan Xiao and Jun-Ming Hu were incorrectly published as Hai-Juan Xiao and Jun-Ming Hou. These errors were introduced during typesetting and thus the publisher apologizes for this error, and acknowledges them as such in this Correction article.

The original article has also been updated to reflect this correction.
